# First person – Vlasta Lungova

**DOI:** 10.1242/dmm.049746

**Published:** 2022-08-23

## Abstract

First Person is a series of interviews with the first authors of a selection of papers published in Disease Models & Mechanisms, helping early-career researchers promote themselves alongside their papers. Vlasta Lungova is first author on ‘ Exposure to e-cigarette vapor extract induces vocal fold epithelial injury and triggers intense mucosal remodeling’, published in DMM. Vlasta is a scientist in the lab of Susan L. Thibeault at the University of Wisconsin-Madison, WI, USA, investigating the mechanisms of laryngeal and vocal fold mucosal development and postnatal regeneration.


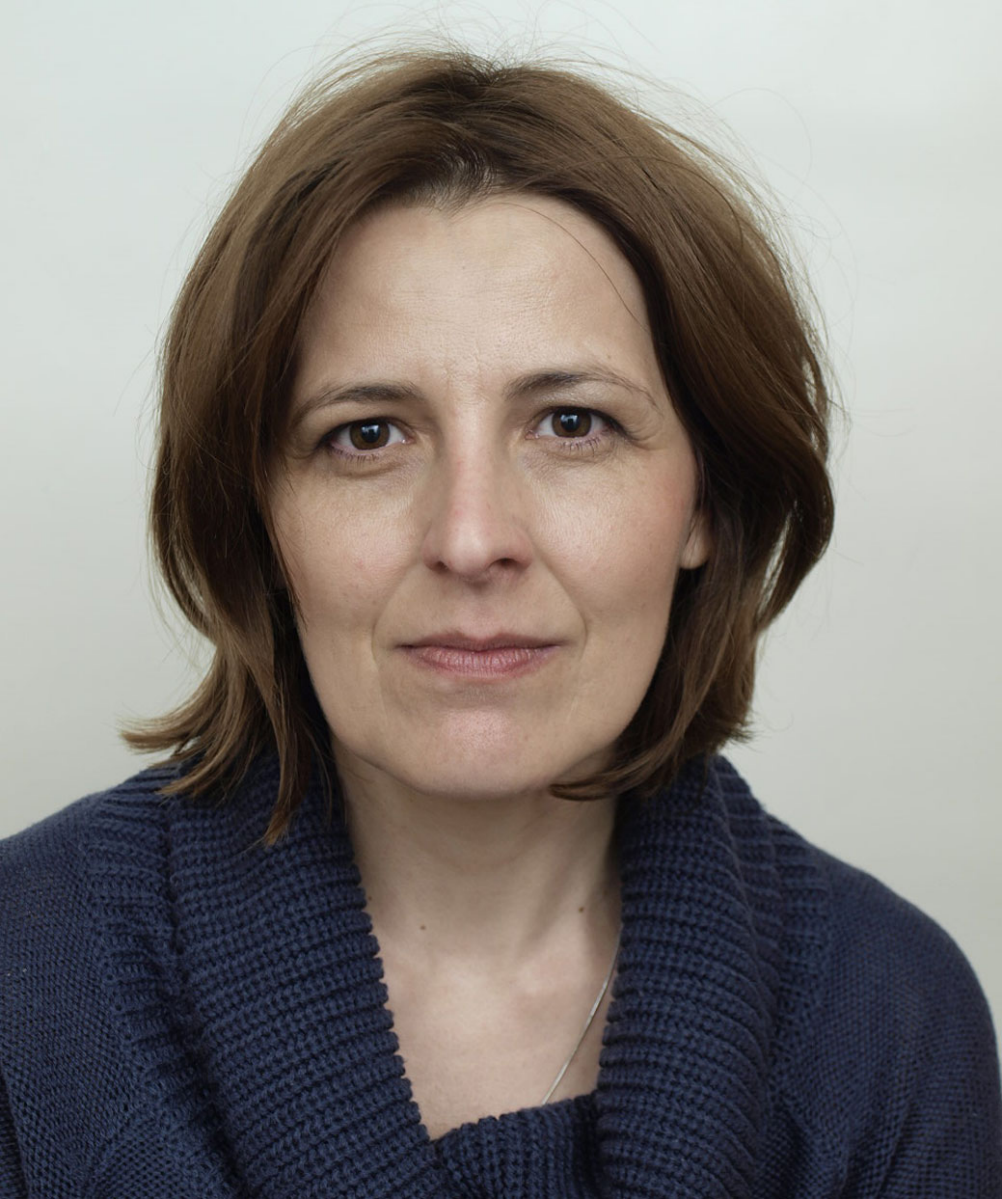


**Vlasta Lungova**

**How would you explain the main findings of your paper to non-scientific family and friends?**

In this study, we evaluated the possible consequences of vaping on human vocal fold mucosal structure and function during electronic cigarette (e-cig) vapor exposure and subsequent mucosal regeneration. Vaping is an emerging phenomenon that is becoming very popular worldwide and is linked to e-cig- or vaping-associated lung injuries (EVALI) and a number of deaths. Besides the lungs and other vital organs, vaping also affects the laryngeal and vocal fold (VF) mucosae, which are involved in voice production, and as parts of the conducting airways, they are directly exposed to inhaled vaporized e-liquids. A recently reported case of epiglottitis in an adolescent female patient revealed a connection between vaping and swollen laryngeal structures that seriously impaired voicing and breathing and required hospitalization. The clinical course and biopsy findings showed signs of direct chemical epithelial injury, which led to a subsequent inflammatory reaction. In our study, we aimed to recapitulate this phenomenon in *in vitro* conditions.

We found that exposure of engineered human VF mucosae to 0.5% and 5% e-cig vapor extracts for 1 week induced chemical epithelial injury, likely caused by defective lipid metabolism and inefficient clearance of lipid/solvent particles, such as medium-chain fatty acids, that gathered in the cytoplasm and intercellular spaces. Next, we demonstrated that the lipid-mediated VF mucosal injury induced moderate mucosal inflammation and triggered intense epithelial remodeling associated with enhanced accumulation of P63-positive basal cells, increased cytokeratin 14, and laminin subunit α-5 depositions. These reactive epithelial changes may lead to basal cell hyperplasia, hyperkeratinization and local basement membrane thickening, which may alter VF mucosal properties and present a consequential threat to VF mucosal health and function.

“Creating an *in vitro* model of human VF mucosae by our lab was extremely valuable for the field of laryngology.”

**What are the potential implications of these results for your field of research?**

There are several practical implications of our results for the laryngology field and for research in general. First, we performed transcriptome analysis of genes related to human fatty acid metabolism in human VF mucosal cells exposed to e-cig vapor extracts. We found significant dysregulation in the transcription of genes involved in medium-chain fatty acid degradation in human VF mucosal cells exposed to commercially available e-liquids with nicotine or with nicotine and flavor. This finding supports the fact that lipid aggregates that accumulate in the cell cytoplasm likely contain medium-chain fatty acids. Since medium-chain fatty acids can pass freely through the cell membrane, they represent a major risk factor associated with vaping, along with vitamin E acetate. We appeal for wide testing of commercial e-cig products to identify potential risks to e-cig users’ health.

Next, the VF epithelial responses measured in this study during the regeneration phase resemble the behavior of VF epithelial cells in response to repeated irritations and/or in response to VF injury, and appear to be adaptive mechanisms that replace susceptible epithelia with more resistant ones. However, these adaptive changes may have adverse consequences on VF mucosal health, as they may impair epithelial transport efficiency and increase the cell mass, altering VF vibration and voicing. Moreover, similar histopathological features such as basal cell hyperplasia and hyperkeratinization have also been found in benign and neoplastic VF lesions. Future clinical studies are necessary to confirm whether e-cig users are more susceptible to VF diseases compared to conventional cigarette smokers and non-smokers.

Lastly, in this study, we provided a better understanding of the immunomodulatory consequences of e-cig vapor exposure and provided the foundation for further investigation focusing on the mechanisms used by damaging events to activate VF innate responses, in order to identify potential therapeutic targets for modulating VF mucosal inflammation.

**What are the main advantages and drawbacks of the model system you have used as it relates to the disease you are investigating?**

To study the effect of vaping on human VF mucosal cells, we used our recently developed model of human induced pluripotent stem cell (hiPSC)-derived VF mucosae. Contrary to previously published studies using *in vitro* human-sourced VF mucosae, this system uses an unlimited source of hiPSC-derived VF epithelial cells, which are differentiated *in vitro* and follow their embryonic development. Throughout their differentiation, they are re-seeded on collagen-fibroblast constructs that mimic the lamina propria. As reported previously, the resulting engineered VF mucosae resemble the native human VF mucosae with a well-developed P63^+^ basal cell layer, a well-defined laminin subunit α-5 basement membrane and layers of suprabasal cells that re-establish stratification. These features are critical for *in vitro* VF mucosal reconstruction and its utilization for translational or pharmacological purposes. The main drawback of this system lies in the fact that this model contains only two main cell types: hiPSC-derived VF epithelial cells and primary VF fibroblasts. In this study, we evaluated the inflammatory cell responses and expression levels of cytokines/chemokines without the presence of immune cells and macrophages, which can influence cytokine/chemokine transcript levels. We understand that further validation of our *in vitro* system and cultivation of VF mucosal cells with a population of immune cells is necessary to provide a comprehensive analysis of the effects of e-cig vapor extract on VF mucosal inflammation.

**Figure:**
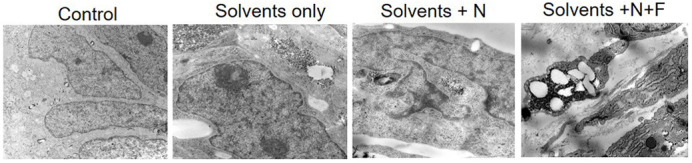
**Lipid-mediated vocal fold epithelial injury caused by exposure to e-cigarette vapor extracts.** The ultrastructure of apical cells in the control group shows tightly packed cells. However, the ultrastructure of apical cells in vocal fold mucosae exposed to e-liquids with solvents only, e-liquids with nicotine (N) and nicotine with flavor (N+F) show lipid/solvent aggregates that accumulate in the cytoplasm and intercellular spaces, causing them to slough off the cell surface and induce lipid-mediated epithelial injury.

**What has surprised you the most while conducting your research?**

Creating an *in vitro* model of human VF mucosae by our lab was extremely valuable for the field of laryngology. It was very encouraging to see that hiPSC-derived VF epithelial cells are functional and capable of acting differently in response to distinct environmental insults. As reported previously, upon exposure to conventional cigarette smoke extract, we noticed elevated levels of pro-inflammatory genes accompanied by increased mucus production and keratotic structural changes in the VF epithelium, where cytokeratin 14 preferentially accumulated in apical cell layers along with cytokeratin 13. Clinically, the smoking status can be associated with chronic laryngeal inflammation, increased mucus expression and keratotic VF lesions. On the other hand, in this study, we have shown that e-cig vaping exposure caused lipid-mediated VF chemical epithelial injury, which triggered VF mucosal reparative processes and activated VF mucosal immune responses, similar to what was reported in animal and/or human case studies. These findings suggest that the hiPSC-derived model of human VF mucosae can provide valuable information about VF mucosal cell abnormal behavior in response to environmental challenges, and holds great promise for the investigation of toxicity and mechanisms of tobacco- or vaping-related cellular injuries/pathologies in human VF stratified epithelia.

**What do you think is the most significant challenge impacting your research at this time and how will this be addressed over the next 10 years?**

I think that the development of engineering-based therapeutics for the prevention and treatment of VF diseases, including VF scarring, and the development of engineered VFs for tissue replacement therapies present the major challenges in our field. To achieve these goals, it is necessary to gain a fundamental understanding of VF cellular heterogeneity, relationships between distinct cell populations and mechanistic insights into their reciprocal interactions in healthy, injured and diseased vocal folds. There are several ways future voice research can address these challenges over the next 10 years. First, the utilization of cutting-edge single-cell and spatial sequencing technologies can provide an unbiased characterization of VF cell diversity and spatial organization in *in vivo* animal models and in humans. Next, improved engineering-based organotypic three-dimensional VF cell cultures along with VF organoid models will enable experimental manipulation of genes and signaling pathways involved in VF reparative and disease processes in an *in vitro* setting. It will be highly beneficial if these *in vitro* studies were supported by data obtained from human patients. Lastly, the incorporation of mouse/rodent transgenic models will enable the genetic manipulation of genes of interest *in vivo* to complement human data and provide a cross-species comparison.

“Early-career scientists need to use every opportunity to talk to faculty members and learn from them […]”

**What changes do you think could improve the professional lives of early-career scientists?**

The Department of Surgery, University of Wisconsin-Madison offers peer support, and career-development seminars. I work as a scientist, which is a non-tenure track position associated with job instability and is highly dependent on funding. I am grateful that I had an opportunity to attend a grant-writing course organized by the Department of Medicine, UW Madison to improve my grant-writing skills and closely cooperated with my mentor, the PI, while submitting my first research grant.

I think that early-career scientists need to use every opportunity to talk to faculty members and learn from them, either directly through mentor-mentee relationships or by attending different courses or seminars offered by their institution. Attending scientific conferences also helps as they usually incorporate career-development discussion forums into their programs, selecting experts from both academia and industry. I will also welcome more funding opportunities for early-career researchers, especially in the research areas that are underestimated and in which it is difficult to get funding.

**What's next for you?**

In the current paper, I studied the responses of VF mucosal cells to e-cig vapor exposure and consequent VF regeneration. My next step will be to improve our organotypic model of human VF mucosae by adding immune cells into the system and investigating the relationship between healthy versus compromised VF epithelial barriers that are simultaneously colonized by commensal and/or pathogenic bacteria. I would like to focus on the mechanisms bacteria use to invade the epithelium and activate VF mucosal immune responses to study VF mucosal inflammatory disorders.
